# Kinesio Taping Does Not Alter Quadriceps Isokinetic Strength and Power in Healthy Nonathletic Men: A Prospective Crossover Study

**DOI:** 10.1155/2015/626257

**Published:** 2015-12-24

**Authors:** Paweł Korman, Anna Straburzyńska-Lupa, Radosław Rutkowski, Jakub Gruszczyński, Jacek Lewandowski, Marcin Straburzyński-Lupa, Dawid Łochyński

**Affiliations:** ^1^Department of Physiotherapy, University School of Physical Education, 61871 Poznan, Poland; ^2^College of Health, Beauty and Education in Poznan, 60133 Poznan, Poland; ^3^College of Education and Therapy, 61473 Poznan, Poland; ^4^Department of Motor Rehabilitation, University School of Physical Education, 61871 Poznan, Poland

## Abstract

*Objectives*. The effects of Kinesio Taping (KT) on muscular performance remain largely unclear. This study aimed to investigate the acute effects of KT on the maximum concentric and eccentric quadriceps isokinetic strength.* Study Design*. This is a single-blinded, placebo crossover, repeated measures study.* Methods*. Maximum isokinetic concentric/eccentric extension torque, work, and power were assessed by an isokinetic dynamometer without taping (NT) and with KT or placebo taping (PT) in 17 healthy young men. Repeated measures one-way analysis of variance (ANOVA) was used for statistical analyses.* Results*. Testing concentric contractions at 60°/s or 180°/s isokinetic speed, no significant differences in peak torque (Nm), total work (J), or mean power (W) were noted among the application modes under different conditions. Testing eccentric contractions at 30°/s or 60°/s isokinetic speed, no significant differences in mentioned parameters were noted, respectively. KT on the quadriceps neither decreased nor increased muscle strength in the participants.* Conclusion*. KT application onto the skin overlying the quadriceps muscle does not enhance the strength or power of knee extensors in healthy men.

## 1. Introduction

Physical solutions aimed at improving muscle contractile performance for sports and rehabilitation purposes are immensely important. Recently, the novel modality “Kinesio Taping” (KT), developed by the Japanese chiropractor Kenzo Kase [1], has received substantial attention [2–4]; it is widely used in various sports disciplines, for example, athletics, tennis, and volleyball, to prevent musculoskeletal injury and restore, maintain, or facilitate functional performance. There are several hypotheses regarding the neuromuscular effects of KT. KT application is assumed to provide a constant shear force on the skin [5], improve proprioception through increased stimulation of cutaneous mechanoreceptors [6], and restore correct muscle function by supporting weakened muscles [1]. However, reports concerning the effects of neuromuscular KT applications show conflicting results, with only few studies demonstrating the beneficial effects of KT on muscle contractile performance. For example, Murray [6] demonstrated enhanced electromyographic (EMG) activity of the quadriceps muscle after KT application in patients in the postoperative phase after anterior cruciate ligament repair. Vithoulka et al. [7] reported an increase in the peak eccentric torque in healthy nonathletic individuals after KT application on the quadriceps. Furthermore, some studies demonstrated the positive effects of KT (increased muscle strength) on different muscles, such as biceps brachii [5] and gastrocnemius [8]. In contrast, Fu et al. [9] and Vercelli et al. [10] demonstrated that, in healthy people, the concentric and eccentric muscle strength of the quadriceps and hamstrings were not influenced by KT. Similarly, Lins et al. [11] revealed that KT application on the quadriceps was not capable of altering lower limb function, one-footed static balance, or peak knee extensor torque in healthy women.

However, owing to serious methodological inconsistencies in the manner of placebo taping, the results of previous studies are difficult to interpret. For example, Vithoulka et al. [7] and Fratocchi et al. [5] used sham taping across muscle fibres with no explanation for this decision. Such inconsistencies can cause issues in reference values, leading to ambiguities in the results. In the light of the scarcity of equivocal research assessing the effects of KT on muscular performance, we aimed to investigate the acute effects of KT on the maximum concentric and eccentric quadriceps isokinetic strength. As suggested by Kase et al. [1], we assumed that the application of the stretched tape onto elongated muscle would facilitate muscle contraction in both the concentric and eccentric modes. Therefore, we hypothesised that KT application would increase quadriceps strength and power. To the best of our knowledge, the muscle power has not been tested in this aspect previously. Enhancing dynamic strength performance of thigh muscles through the application of KT could serve for prophylactic purposes to decrease the risk of knee joint overuse or injury during recreational activities imposing high mechanical stress on this joint.

## 2. Methods

Seventeen healthy males were recruited by convenient sampling from university students (see Table 1).

Subjects were informed that they could end a testing session at any time without penalty. None of the subjects were aware of any neuromuscular or orthopaedic disorder affecting their dominant leg. Participants were moderately active; however, none were involved in strength-type or endurance-type lower limb training programs at least six months before study enrolment. They received detailed explanations about the aims, benefits, and risks involved with this investigation. Participants were not aware of potential taping benefits. Written informed consent was obtained from all the participants, and this study was conducted in accordance with the Declaration of Helsinki and approved by the Ethical Committee, Medical University of Poznan.

To verify the effects of KT taped over the quadriceps in its maximum strength capabilities, three conditions were tested: no taping (NT), Kinesio Taping (KT), and placebo taping (PT). The two tapes used in the study included the highly stretchable (up to 75% of its original length) latex-free cotton tape with heat-activated acrylic adhesive (Kinesio Tape, KT) (K-Active, Nitto Denko, Japan) [1, 3] and a nonelastic medical tape (PT) (Polovis PLUS, 3M, Poland).

**Figure 1:**
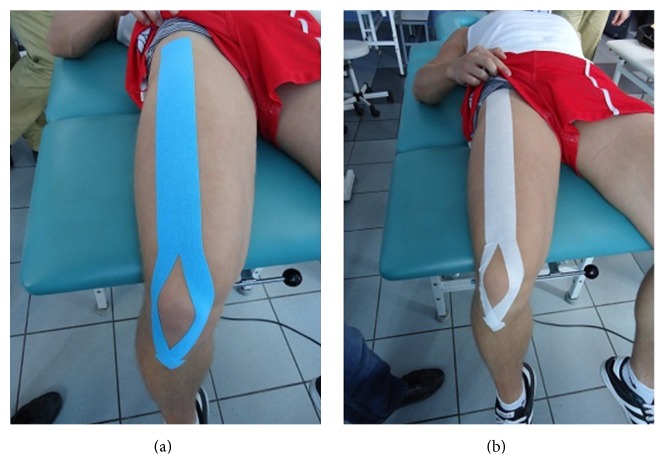
The taping method and the subject's posture when applying KT (a) and PT (b).

The participants were laid in a supine position with the knee flexed at 90°. KT and PT were taped over the skin area covering the quadriceps femoris muscle of the dominant lower limb. Both applications were Y-shaped; each started from the anterior inferior iliac spine, bisected at the junction between the quadriceps femoris tendon and the patella, and ended at the tibial tuberosity (Figure 1). The distance between both anatomical points was measured for each subject to set the length for each application. While the length of PT was kept constant, the KT was stretched to 120% of its original length to cover this distance.

Maximum isokinetic concentric/eccentric extension torque, work, and power were assessed by an isokinetic dynamometer (Isoforce, Tur Therapietechnik GmbH, Germany). Only the dominant leg, determined using the leg dominancy test, was evaluated [12]. Only two persons were left leg dominant. Participants were comfortably seated in an adjustable chair and the upper body and opposite thigh were secured with straps. The position of the hips was set at 100° of flexion and the testing knee was aligned with the axis of the dynamometer's lever arm. The resistance adapter of the dynamometer was secured to the leg 5 cm above the malleolus line. The testing range of motion was set at 0–90°, and 0° was defined as the knee at neutral extension position (anatomical zero). Participants performed two sets of three concentric/eccentric contractions at slow (60° immediately followed by 30°/s) and fast (180°/s immediately followed by 60°/s) isokinetic speeds in three different conditions, that is, NT, KT, and PT. They were verbally encouraged to produce maximum effort during performance. The orders of the conditions and sets were randomised for each participant. The allocation sequence was obtained by a computer-generated list of random numbers using an online research randomizer (http://www.randomizer.org/). To prevent fatigue, testing conditions were separated with 15-minute rests. Two-minute breaks were introduced between the sets, and 1-minute break intervals were implemented between particular movement repetitions. A 5-minute ride on a stationary bicycle and several submaximum repetitions of movement at a preset speed served as warm-up and preceded maximum isokinetic testing. The calibration of the dynamometer was performed before each testing session.

The peak torque expressed in Nm, the maximum total work defined as the area under the torque-time curve and expressed in Joules, and the average power defined as the total work divided by time and expressed in Watts were obtained from the 3 trials of concentric and eccentric contractions of the knee extensors.

All statistical analyses were performed by individuals blinded to group assignment. The data were analysed using Statistica 8.0 package. Means ± SD were calculated for each parameter studied. Repeated measures one-way analysis of variance (ANOVA) was used to detect differences among the three taping applications. All effects were considered significant at the 0.05 significance level.

## 3. Results

**Figure 2:**
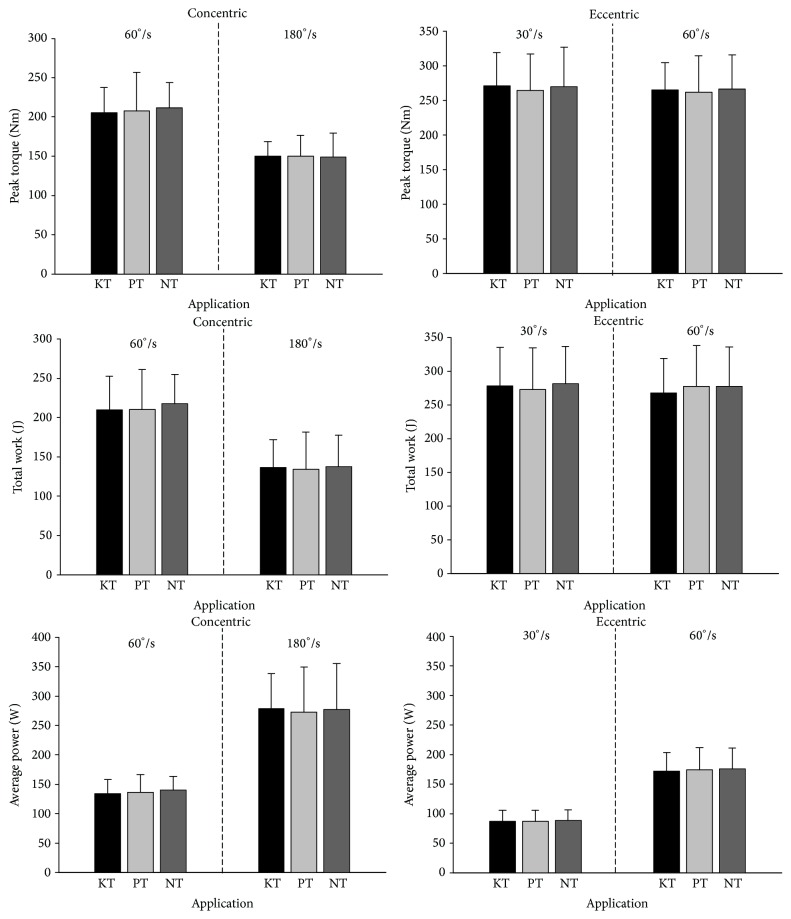
Concentric and eccentric isokinetic variables. KT: Kinesio Tape; PT: placebo tape; NT: no tape. Values are expressed in mean ± SD.

One-way analysis of variance did not reveal any significant effects of KT or PT for concentric contractions at 60°/s or 180°/s isokinetic speed on the quadriceps peak torque (*F*(2,17) = 0.353, *p* = 0.705; *F*(2,17) = 0.027, *p* = 0.973), total work (*F*(2,17) = 0.443, *p* = 0.646; *F*(2,17) = 0.114, *p* = 0.893), or average power (*F*(2,17) = 0.560, *p* = 0.576; *F*(2,17) = 0.105, *p* = 0.901) (Figure 2). Similarly, no real difference was noted between the tested conditions for eccentric contractions at 30°/s or 60°/s isokinetic speed on the quadriceps peak torque (*F*(2,17) = 0.207, *p* = 0.814; *F*(2,17) = 0.139, *p* = 0.871), total work (*F*(2,17) = 0.459, *p* = 0.636; *F*(2,17) = 0.639, *p* = 0.534), and average power (*F*(2,17) = 0.166, *p* = 0.848; *F*(2,17) = 0.147, *p* = 0.864).

## 4. Discussion

Our results suggest that KT did not significantly improve muscle peak torque, power, or work in healthy nonathletic males. In addition, PT application also did not influence quadriceps performance. These results were consistent with those by Fu et al. [9] who found no effects of KT on the quadriceps peak torque or total work in 14 healthy men. Similarly, Janwantanakul and Gaogasigam [13] could not provide any evidence that KT affected EMG activity. Furthermore, Lins et al. [11] found no significant differences in the EMG activity of the vastus lateralis or the concentric and eccentric knee peak torque at 60°/s. Interestingly, Wong et al. [14] also confirmed the lack of the influence of KT on peak torque and total work; however, they noted that the time to peak torque of extension was significantly shortened with KT application onto the vastus medialis.

In contrast, only few studies have indicated a positive effect of KT application on quadriceps performance. The results presented by Vithoulka et al. [7] suggest that KT application in 20 healthy women on the anterior surface of the thigh may increase isokinetic eccentric peak torque. Furthermore, improved quadriceps strength at 60°/s and 180°/s and static and dynamic balance scores before and at 45 min after KT application were reported by Aytar et al. [15]. It is worth mentioning that KT applications in these studies differed from the method proposed by Kase, with KT covering a larger area. This may be explained by the hypothesis that the stimulus needs to be great enough to influence the muscle. Some authors [1, 16] have explained this as the mechanical effects of KT on fascia or its sensory tactile effects on the skin.

One factor that can explain the lack of alterations in the muscle performance after KT application in our study was the inclusion of healthy subjects. They had no neuromuscular pain or dysfunctions that could be minimised by applying KT [17], which could facilitate muscle performance [6]. Moreover, the hypothesis that KT application produces an increase in the interstitial space improving blood flow and possibly favouring a rise in muscle activation also was not proven [11].

It is worth mentioning that power, which was examined in this study, did not change significantly; this point has not been mentioned previously. Power is the rate of performing work, that is, the product of force and velocity [18]. This value is considered as one of the main determinants of athletic performance that require the explosive production of force, such as jumping and throwing [19].

Our study has certain limitations. Our results reveal only the acute effects of KT application; therefore, we cannot comment on the positive effects of prolonged KT application on muscle strength. Furthermore, we cannot exclude the possibility that the stretching applied in the KT application in this study was too weak to facilitate the stronger contraction of the muscle.

## 5. Conclusion

We demonstrated that acute KT application onto the skin overlying the quadriceps muscle did not alter peak torque, total work, or average power in young healthy men. The application of KT immediately before physical effort, as performed in this study, does not improve muscle performance. It can be assumed that KT applied in this study onto thigh muscles is ineffective in improving muscle strength and hence presumably does not enhance knee joint protection by dynamic stabilization. Since our results are limited to active young healthy men engaging in recreational physical activity, further studies are warranted to investigate the physiological and other effects of KT application and its underlying mechanisms.

## Figures and Tables

**Table 1:** Demographic data of the studied population of subjects.

Parameter	Mean	SD
Age (years)	24.3	0.6
Weight (kg)	74.9	7.4
Height (cm)	178.6	5.7
BMI (kg/m^2^)	23.5	1.5
